# Resveratrol Protects Mouse Oocytes from Methylglyoxal-Induced Oxidative Damage

**DOI:** 10.1371/journal.pone.0077960

**Published:** 2013-10-23

**Authors:** Yu Liu, Xiao-Qin He, Xin Huang, Lu Ding, Lin Xu, Yu-Ting Shen, Fei Zhang, Mao-Bi Zhu, Bai-Hui Xu, Zhong-Quan Qi, Hai-Long Wang

**Affiliations:** 1 Organ Transplantation Institute, Medical College, Xiamen University, Xiamen City, Fujian Province, China; 2 Department of Gynaecology and Obstetrics, Zhongshan Hospital, Xiamen University, Xiamen City, Fujian Province, China; 3 Center of Reproductive Medicine, Xiamen Maternity and Child Health Care Hospital, Xiamen City, Fujian Province, China; Institute of Zoology, Chinese Academy of Sciences, China

## Abstract

Methylglyoxal, a reactive dicarbonyl compound, is mainly formed from glycolysis. Methylglyoxal can lead to the dysfunction of mitochondria, the depletion of cellular anti-oxidation enzymes and the formation of advanced glycation ends. Previous studies showed that the accumulation of methylglyoxal and advanced glycation ends can impair the oocyte maturation and reduce the oocyte quality in aged and diabetic females. In this study, we showed that resveratrol, a kind of phytoalexin found in the skin of grapes, red wine and other botanical extracts, can alleviate the adverse effects caused by methylglyoxal, such as inhibition of oocyte maturation and disruption of spindle assembly. Besides, methylglyoxal-treated oocytes displayed more DNA double strands breaks and this can also be decreased by treatment of resveratrol. Further investigation of these processes revealed that methylglyoxal may affect the oocyte quality by resulting in excessive reactive oxygen species production, aberrant mitochondrial distribution and high level lipid peroxidation, and resveratrol can block these cytotoxic changes. Collectively, our results showed that resveratrol can protect the oocytes from methylglyoxal-induced cytotoxicity and this was mainly through the correction of the abnormity of cellular reactive oxygen species metabolism.

## Introduction

Methylglyoxal (MG), a highly reactive dicarbonyl compound, is a metabolic byproduct of glucose[1]. MG is mainly derived from glycolysis[2] and increasing evidence reveals that MG can be generated by degradation of carbohydrates in foods and beverages such as soy sauces and coffee[3], [4]. MG attacks the arginine, lysine and cysteine residues of proteins to form irreversible advanced glycation end products (AGEs), which subsequently leads to more cross-linking and degradation of proteins[5]. This accumulation of AGEs is considered to be responsible for the long-term complications of diabetes and ageing[6], [7]. In addition, MG contributes to the cytotoxicity, causing cell damages like apoptosis[8]. MG-induced apoptosis occurred through mitochondrion-dependent processes and the imbalance of cellular redox state[9]. Recently, two studies focused on the cytotoxicity of MG on the female reproductive function, especially on the oocytes[10], [11]. Oocytes maturation can be seriously disturbed by the MG-induced breakage of DNA, disruption of spindle assembly, depletion of anti-oxidation enzymes and disorder of mitochondrial function. All of these may root in the elevation of intracellular reactive oxygen specie (ROS) level and this implies that a supplement of anti-oxidant may counteract the deleterious effect of MG on oocytes.

Resveratrol (3, 5, 4′-trihydroxystilbene) is a common phytoalexin which belongs to the large family of biologically active substances existing in peanuts, Itadori tee, skin of grapes and red wine[12]. Mounting evidence suggests that resveratrol could act as a powerful anti-cancer[13], anti-inflammatory[14], anti-diabetes[15] and anti-oxidation[16] agent. Moreover, resveratrol functions as a potent SIRT1 activator which is capable of mimicking the effects of calorie restriction and regulating longevity in lower organisms[17]. Most strikingly, resveratrol can exert a powerful anti-oxidation effect in organisms. This effect can be illustrated by its well-recognized effect to the low density lipoprotein oxidation[18]. Meanwhile, resveratrol scavenges cellular ROS and corrects radical-induced responses such as DNA damage[19], imbalance of mitochondria redox state[20], inactivation of cytochrome P450[21] and interfering cellular signal transductions[22].

We hypothesized that supplement of resveratrol could counteract MG-induced cytotoxicity in mouse oocytes. In this study, oocytes were divided into three groups: control, MG-treated and resveratrol-MG-treated. We examined DNA double-strand break (DSBs), the rates of *in vitro* oocyte maturation (IVM), spindle assembly, chromosome congression, intracellular ROS level, mitochondrial distribution and lipid peroxidation level. Our results showed that resveratrol could reverse the adverse effect of MG on mouse oocytes.

## Materials and Methods

### Ethic statement

Mice care and use were conducted in strict accordance with the recommendations in the Guide for the Care and Use of Laboratory Animals of the National Institutes of Health. The protocol was approved by Animal Studies Committee of Xiamen University, China (approval ID: XMUMC 2011-10-08). ICR mice were housed and bred at temperature-controlled room, received the standard murine chow diet, and kept on a cycle of 12 h light and 12 h dark, with the darkness starting from 19:00. The mice were killed by cervical dislocation. All efforts were made to minimize suffering and the only procedures performed on the dead animals were getting ovaries and the collection of oocytes from the ovaries.

### Methyglyoxal and resveratrol treatment

Methyglyoxal (Sigma, USA) was dissolved in phosphate buffered saline (PBS) and stored at a stock concentration of 100 mM. Resveratrol (Sigma, USA) was dissolved in dimethylsulfoxide (DMSO) and stored at a stock concentration of 50 mM. The MG stock solutions were serially diluted in M2 medium (Sigma, USA) to working concentration of 75 µM. The resveratrol stock solutions were diluted in M2 medium plus MG to working concentration of 5, 10 and 25 µM with a final DMSO content which is below 0.1% in the medium (v/v).

### Oocyte collection and culture

Fully grown, germinal vesicle (GV)-intact oocytes were retrieve by puncturing antral follicles with sterile needles and washed thoroughly with M2 medium. Cumulus cells surrounding the oocytes were removed by gentle pipetting through a narrow-bore glass pipette. Denuded oocytes were cultured in M2 medium with or without 75 µM MG and different concentration of resveratrol to specific stages under mineral oil at 37°C in a humidified atmosphere of 5% CO_2_ in air. GV-stage oocytes which were stained with γ-H2AX or MitoTracker Red were cultured for 6 h in the presence of 2.5 µM milrinone (Sigma, USA). Metaphase I (MI)-stage and metaphase II (MII)-stage oocytes were cultured in M2 medium for 8.5 h and 12 h.

### Immunofluorescence and confocal microscopy

The protocol was basically the same as described in our previous studies[23]–[25]. Oocytes were fixed with 4% paraformaldehyde for 30 min. After being permeabilized with 0.5% Triton X 100 at room temperature for 30 min, oocytes were blocked in 1% bull serum albumin (BSA)-supplemented PBS for 1 h and then incubated overnight at 4°C with 1∶100 rabbit anti-γ-H2AX antibody (Abcam, USA), 1∶30 sheep polyclonal to BubR1 antibody (Abcam, USA) or 1∶200 mouse anti-α-tubulin-FITC antibody (Sigma, USA), respectively. After three washes in PBS containing 0.1% Tween 20 and 0.01% Triton X-100 for 5 min each, the oocytes were labeled with Donkey Anti-Sheep IgG Antibodies Conjugates Alexa Fluor 546 (Invitrogen, USA, 1∶250, to stain BubR1) or Donkey Anti-Rabbit IgG Antibodies Conjugates Alexa Fluor 546 (Invitrogen, USA, 1∶800, to stain γ-H2AX) for 1 h at room temperature. Then after three washes in PBS containing 0.1% Tween 20 and 0.01% Triton X-100 for 5 min each, the oocytes were co-stained and mounted on glass slides with mounting medium with DAPI (Vector, Switzerland). Finally, the oocytes were examined with an FV1000 confocal laser scanning microscope (Olympus, Japan).

### Determination of ROS level

To determine the quantity of ROS production, oocytes were loaded with the oxidation-sensitive fluorescent probe [dichlorofluorescein (DCFH)] by incubation for 30 min at 37°C in M2 medium supplemented with 0.1% DCFH diacetate (DCFH-DA) (Beyotime, China). After three times wash in M2 medium, green fluorescence of oocytes in all group was detected with the FV1000 confocal laser scanning microscope with 450–490 nm (excitation) and 520 nm (emission) filters under the same condition.

### Assessment of mitochondrial distribution

Mitochondrial staining was performed with a MitoTracker Red (Invitrogen, USA) according to the manufacturer's instructions. Briefly, live denuded oocytes were cultured in M2 medium containing 200 nM MitoTracker Red for 30 min at 37°C, 5% CO_2_ in air. After washes, stained oocytes were fixed and viewed by the FV1000 confocal laser scanning microscope.

### Oocyte lipid peroxidation assay

Lipid peroxidation assay was performed with 4, 4-difluoro-5- (4-phenyl-1, 3-buttadienyl)-4-bora-3a, 4a-diaza-s-indacene-3-undecanoic acid 581/591C11 (BODIPY D3861, Invitrogen, USA) according to the manufacturer's instructions. Briefly, oocytes were incubated under oil in 10 µM dye for 30 min at 37°C in 5% CO_2_ in air. The dye loaded oocytes were then washed twice in M2 medium and mounted on slides. Green and red fluorescence of BODIPY were determined using the FV1000 confocal laser scanning microscope.

### Statistical analysis

At least three replicates were conducted and 30–40 oocytes were used for each treatment. Data (mean ± SEM) were analyzed by ANOVA using SPSS software (IBM Corp, USA) followed by the Pearson's chi-square test. The number of oocytes was labeled in parentheses as (n). Difference at *P*<0.05 was considered to be statistically significant and different superscript letters in the figures indicate statistically significant differences.

## Results

### Resveratrol protects against MG-induced DNA breaks

**Figure 1:**
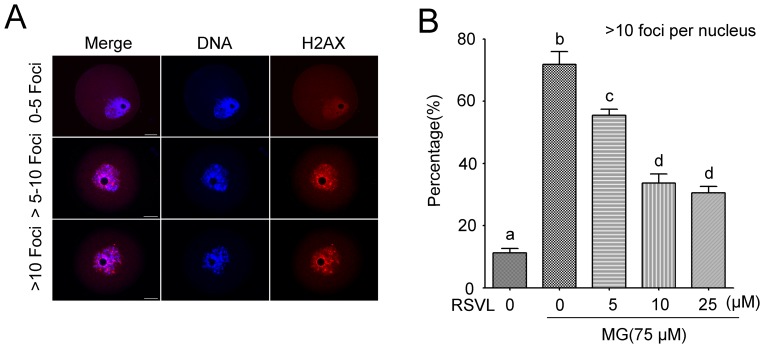
Induction of γ-H2AX foci in GV oocytes following MG and resveratrol (RSVL) treatment. Oocytes were blocked at the GV stage in M2 medium plus 2.5 µM milrinone and were cultured with or without 75 µM MG and different concentration of resveratrol for 6 h. DNA and γ-H2AX were stained and the results show that resveratrol can effectively protect MG-induced the formation of γ-H2AX, the protein marker of DSB. (**A**) Representative samples of control GV-stage oocytes with γ-H2AX 0–5 foci, >5–10 foci and >10 foci. Red, γ-H2AX; Blue, DNA. Scale bar  = 20 µm. (**B**) The proportion of GV oocytes with γ-H2AX >10 foci after MG and different concentration of resveratrol treatment (5, 10 and 25 µM). Data are mean ± SEM of three independent experiments. Different letters denote statistically significant differences (*P*<0.05).

Recent studies have shown that MG may lead to oxidative damage of oocytes via the formation of ROS[10], [11]. ROS may contribute to oxidative stress, resulting in DNA breaks[26]. Therefore we first examined the effects of resveratrol on the DNA damages. We immunoprobed GV oocytes with a γ-H2AX (marker of DNA DSBs) antibody, and found very significant amounts of γ-H2AX foci associated with chromatin in the oocytes incubated with MG. Oocytes with over 10 foci in nucleus obviously increased (**Fig. 1A, B**). However, this MG-induced DNA damage could be prevented by different concentrations of resveratrol and oocytes with high intense γ-H2AX staining significantly reduced in a dose-dependent manner (Control: 11.2±1.5%, n = 121; MG-treated: 71.8±4.1%, n = 120; resveratrol-MG-treated: 5 µM resveratrol: 55.5±2.0%, n = 116; 10 µM resveratrol: 33.7±2.9%, n = 128; 25 µM resveratrol: 30.6±2.0%, n = 112) (**Fig. 1A, B**). This result indicated that resveratrol played a protective role in MG induced DNA damage.

### Effect of MG and resveratrol on oocytes maturation

**Figure 2:**
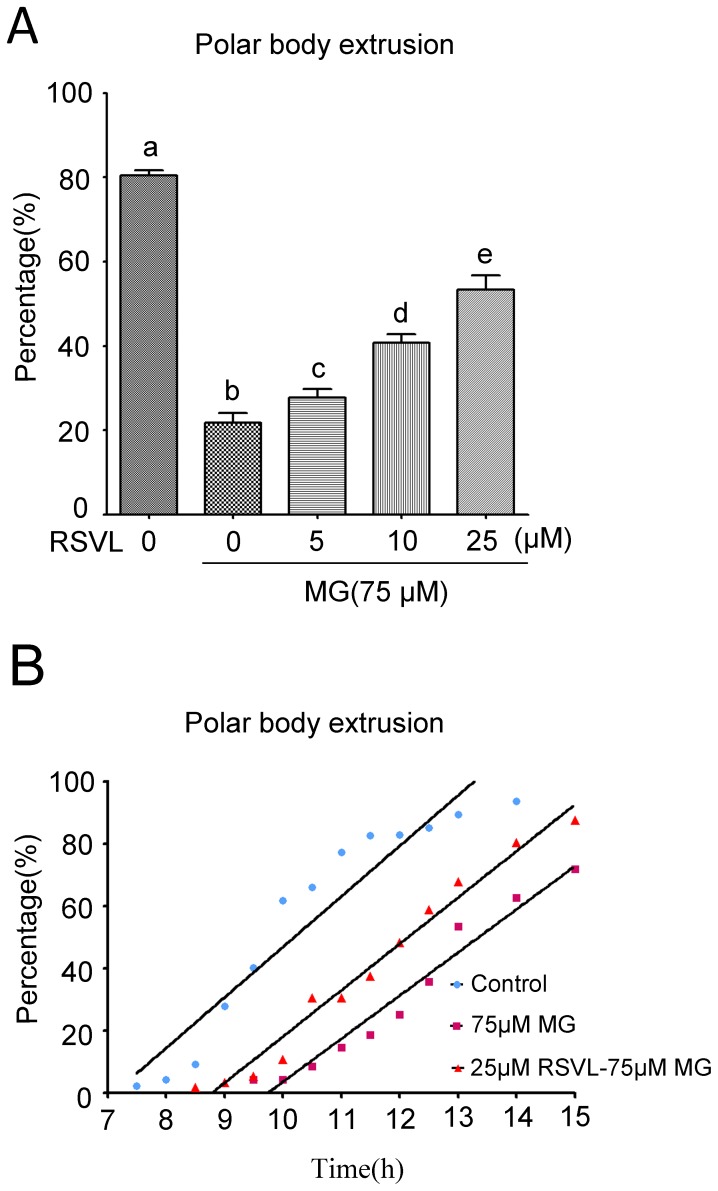
Effect of MG and resveratrol on meiotic maturation of mouse oocytes. (**A**) Percentage of first polar body (PB) extrusion in 75 µM MG and different concentrations of resveratrol group. Different letters denote statistical difference at a *P*<0.05 level of significance. (**B**) Kinetics of PB1 extrusion of control group, 75 µM MG-treated group and 25 µM RSVL-MG-treated group.

We next found that resveratrol rescued MG-induced oocyte maturation delay. Denuded GV oocytes were treated with 75 µM MG for 12 hours, and the percentage of oocytes with extruded first polar body (PB1) was significantly lower than that in control group. We added resveratrol into the culture medium and further found that resveratrol reversely affected MG-induced decrease of PB1 extrusion rate (Control: 80.5±1.2%, n = 207; MG-treated: 21.8±2.3%, n = 221; resveratrol-MG-treated: 5 µM resveratrol: 27.7±2.0%, n = 225; 10 µM resveratrol: 40.8±2.1%, n = 205; 25 µM resveratrol: 53.3±3.4%, n = 227) (**Fig. 2A**). We also quantitatively analyzed the kinetics of PB1 formation and found that resveratrol effectively protected the oocytes from MG-induced PB1 extrusion delay (Linear regression for control group: y = 16.23×−115.5, *p*<0.05; for 75 µM MG group: y = 13.85×−135, *p*<0.05; for 25 µM resveratrol-MG-treated: y = 14.86×−130.9, *p*<0.05; y: rate of PB1 extrusion (%); x: the time of IVM (h)) (**Fig. 2B**).

### Resveratrol prevents MG-induced disturbances in spindle formation and chromosome congression

**Figure 3:**
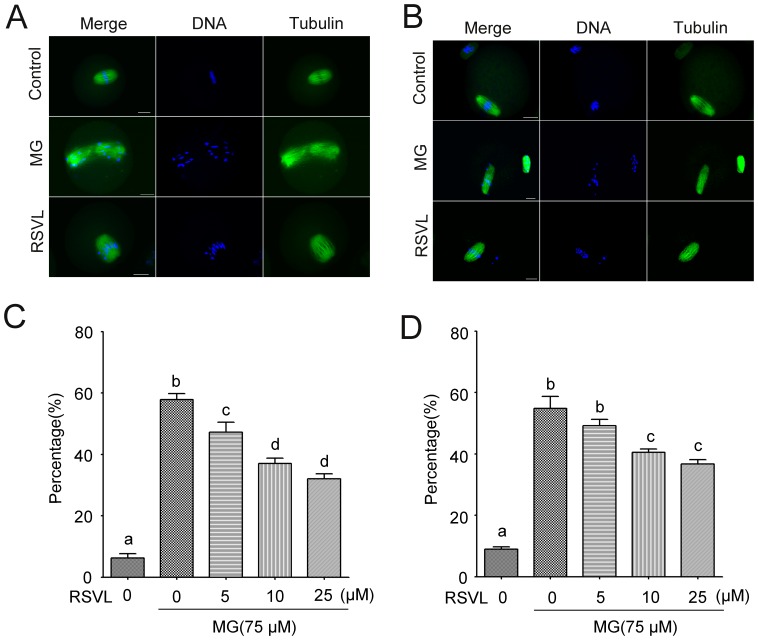
Effect of MG and resveratrol on spindle formation and chromosome alignment of MI and MII oocytes. (**A**) Representative sample of control group, MG-treated group and RSVL-MG-treated group possessing various shapes of MI spindle (green) and chromosomes (blue). Normal spindles displayed a typical shuttle shape and located to the periphery of the oocyte. Chromosomes were aligned exactly on equatorial plate. MG can obviously induce a deformation of spindle and a disturbance of chromosome alignment and this abnormal morphology can be prevented by resveratrol. (**B**) Representative sample of control group, 75 µM MG-treated group and RSVL-MG-treated group possessing various shapes of MII spindle and chromosomes. Scale bar  = 20 µm. (**C**) and (**D**) Percentage of oocytes with abnormal spindle and misaligned chromosomes in MI and MII oocytes from control group, MG-treated group and different concentrations of RSVL-MG-treated group (5, 10 and 25 µM). Data are mean ± SEM of three independent experiments. Different letters denote statistically significant differences (*P*<0.05)

Normal metaphase I (MI) spindles displayed a typical shuttle shape and located to the periphery of the oocyte. Chromosomes were aligned on equatorial plate where they were tightly bound to kinetochore microtubules. The longitudinal axis of the spindle, which traversed the spindle poles, was always perpendicular to the adjacent plasma membrane (**Fig. 3A**). Metaphase II (MII) spindles were found to locate differently in oocytes, but still maintained their shuttle shape appearance. The MII spindle longitudinal axis became nearly parallel to the adjacent plasma membrane (**Fig. 3B**). But after the treatment of MG, we observed the remarkable deformations of MI spindle which tended to elongate. The chromosomes were dispersed throughout the spindle and no obvious metaphase plate was formed. MII spindle was also prone to be longer and thinner and exhibited an abnormal localization (**Fig. 3A, B**). Resveratrol could effectively attenuate these disturbances and more MI and MII oocytes could undergo a correct process of spindle formation and chromosome congression under the resveratrol-treated condition (**Abnormal morphology of MI**: Control: 6.3±1.4%, n = 161; MG-treated: 57.8±2.0%, n = 143; resveratrol-MG-treated: 5 µM resveratrol: 57.8±2.0%, n = 143; 10 µM resveratrol: 37.0±1.8%, n = 149; 25 µM resveratrol: 32.1±1.6%, n = 145. **Abnormal morphology of** **MII**: Control: 9.0±0.7%, n = 145; MG-treated: 54.8±3.9%, n = 145; resveratrol-MG-treated: 5 µM resveratrol: 49.2±2.1%, n = 147; 10 µM resveratrol: 40.5±1.1%, n = 141; 25 µM resveratrol: 36.7±1.4%, n = 150) (**Fig. 3C, D**). This also confirmed that resveratrol could prevent MG-induced oocyte damage.

### Resveratrol prevents MG-induced ROS formation and mitochondrial dysfunction

**Figure 4:**
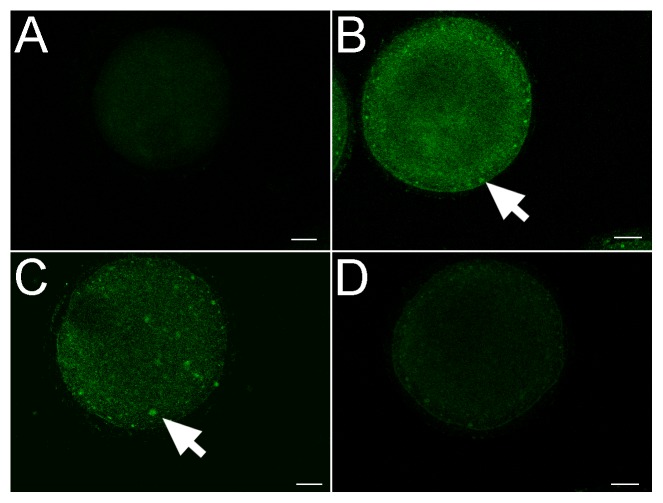
Effect of MG and resveratrol treatment on ROS in oocytes. MI-stage oocytes were incubated in M2 medium plus 0.1% DCFH diacetate (DCFH-DA) for 30 mins. The Effect of MG and resveratrol on ROS production can be measured by the DCFH fluoresence. (**A**) Control group; (**B**) and (**C**) MG-treated group; (**D**) RSVL-MG-treated group. Arrows show the cortex of oocytes with high level of ROS. Scale bar  = 20 µm.

A number of studies have shown that MG triggers oxidative stress in cells[27], [28]. In oocytes, high ROS content may damage DNA and protein, interfere with the metabolism energy sources, alter the lipid fusibility and then inhibit oocyte maturation. Accordingly, we examined whether excessive ROS formation occurs in MG-treated oocytes, and analyzed the effects of resveratrol on ROS formation. DCFH-DA is a high lipid solubility agent which can penetrate through the cell membranes and be hydrolyzed to DCFH. DCFH can be oxidized in cells to be DCF which could be stimulated to green fluorescence emission. Our result revealed the DCFH-DA fluorescence intensity was significantly higher in the oocytes of the MG-treated groups than control; some fluorescence clusters were dispersed in the oocytes suggesting the existence of ROS (**Fig. 4**). And the proportion of oocytes with high fluorescence intensity was higher than that in the control group (**Table. 1**). Treatment with resveratrol dose-dependently attenuated this increase (**Table. 1**), suggesting that resveratrol may play a role of scavenging ROS in oocytes.

**Table 1:** Effect of methylglyoxal and resveratrol on the ROS level and mitochondria distribution of oocytes.

	ROS Level		Mitochondria Distribution			
	n	High level (%)	n(GV)	Abnormal (%)	n(MII)	Abnormal (%)
Control	84	2(2.3±1.2)^#^	95	14(14.6±1.5)^#^	98	16(16.3±1.1)^#^
75 µM MG	78	40(51.9±3.0)	87	45(51.7±2.6)	87	61(70.6±3.0)
5 µM RSVL-75 µM MG	88	39(44.2±1.6)	87	39(44.6±1.3)*	97	56(57.8±2.1)
10 µM RSVL-75 µM MG	80	24(29.5±3.3)	89	30(33.8±2.6)*	89	45(50.3±2.9) *
25 µM RSVL-75 µM MG	95	25(26.3±0.7)*	90	26(28.7±1.6) *	89	41(46.1±2.3) *

* *P*<0.05 and ^#^ *P*<0.05 versus MG group.

**Figure 5:**
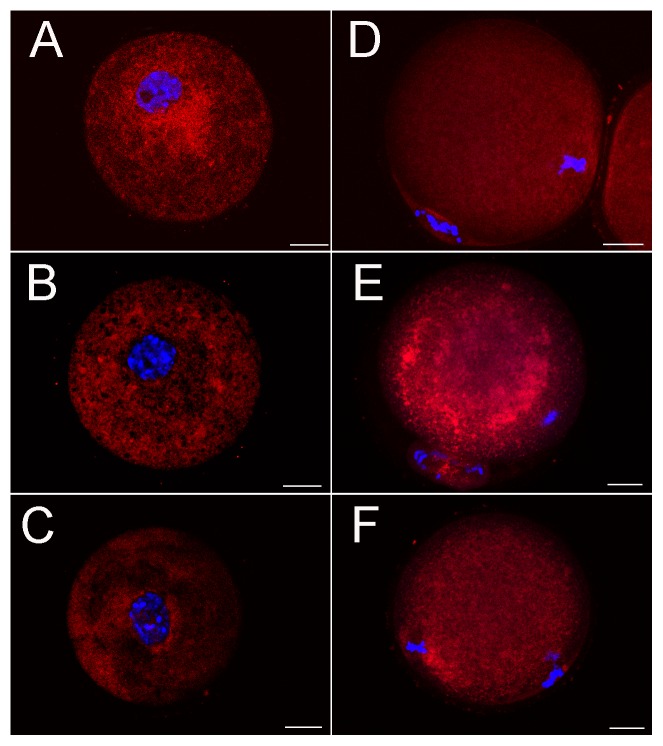
Effect of MG and resveratrol treatment on distribution of mitochondria in meiosis. (**A**) and (**D**) Representative mitochondrial distribution patterns detected by fluorescence microscopy using MitoTracker Red at GV and MII stage in control group. (**B**) and (**E**) Representative mitochondrial distribution patterns at GV and MII stage in MG-treated group. (**C**) and (**F**) Representative mitochondrial distribution patterns at GV and MII stage in RSVL-MG-treated group. Scale bar  = 20 µm.

Suboptimal mitochondria can induce abnormal levels of mitochondrial ATP generation, resulting in chromosomal segregation disorder[29], maturation failure[30] and arrested cell division or abnormal cytokinesis[31]. Meanwhile, dysfunction of mitochondrial may produce an abundance of ROS. Therefore we used the mitochondrial probe to examine the distribution of active mitochondria in oocytes. Our results showed that control GV oocytes displayed a homogeneous mitochondrial distribution pattern or a perinuclear mitochondrial distribution pattern (**Fig. 5A**). However, clustered mitochondria were observed throughout the cytoplasm in MG-treated GV oocytes (**Fig. 5B** **and Table. 1**). This effect could be blocked by treatment of resveratrol (**Fig. 5C** **and Table. 1**). Similar phenomenon was observed in MII oocytes (**Fig. 5D, E, F** **and Table. 1**).

### Resveratrol prevents MG-induced lipid peroxidation

**Figure 6:**
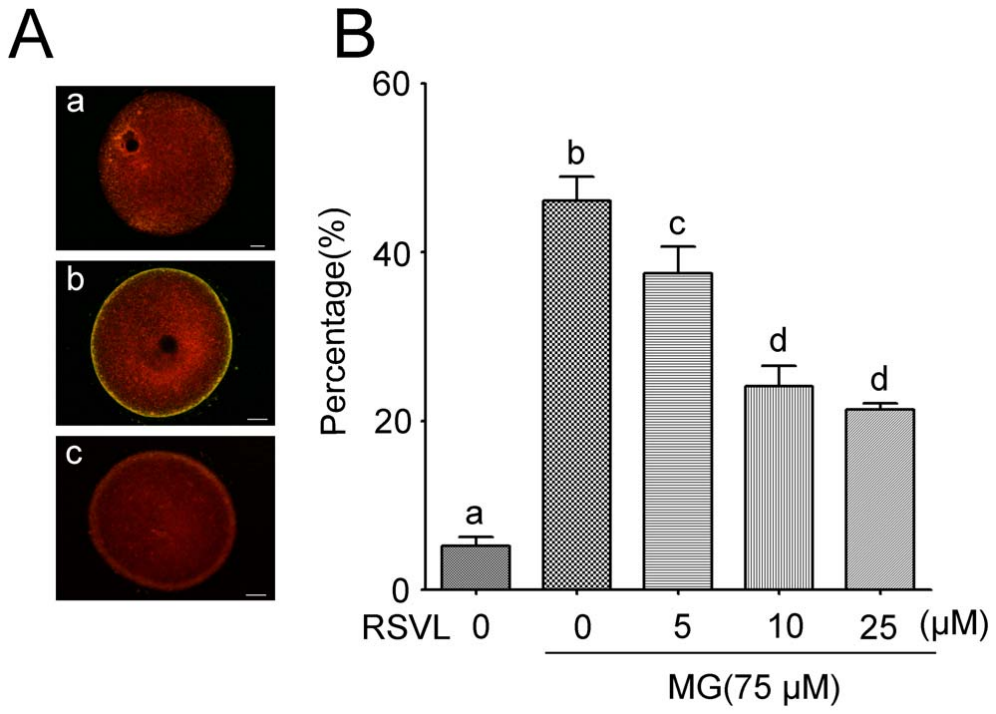
MG and resveratrol-induced oocyte membrane lipid peroxidation in vitro. (**A**) (**a**), (**b**) and (**c**) Representative images of BODIPY-labeled control group, MG-treated group and resveratrol-MG-treated group oocytes. Scale bar  = 20 µm. (**B**) Levels of lipid peroxidation as observed by the of green fluorescence on cortical areas. The bar chart shows the percentage of oocytes with high level of lipid peroxidation from control group, 75 µM MG-treated group and different concentrations of RSVL-MG-treated group (5, 10 and 25 µM). Data are mean±SEM of three independent experiments. Different letters denote statistically significant differences (*P*<0.05).

It has been known that lipid peroxidation generates complex products including hydroperoxides and cleavage products such as malondialdehyde. These products can modify DNA and proteins and exert cytotoxic effects. Besides, the oxidative deterioration of polyunsaturated fatty acid may lose its fusibility and cause the dysfunction of cell membrane. We carried out the lipid peroxidation studies using the fluorescent dye BODIPY. The result revealed significantly increased levels of peroxidative damage in the oolemma membrane of MG-treated oocytes (**Fig. 6A, B**). Meanwhile, treatment with resveratrol significantly alleviated the lipid peroxidation (**Fig. 6C**). Also, the portion of oocytes with a low lipid peroxidation in resveratrol treatment was larger than that in MG group (Control: 5.2±1.0%, n = 95; MG-treated: 46.1±2.8%, n = 89; resveratrol-MG-treated: 5 µM resveratrol: 37.4±3.2%, n = 94; 10 µM resveratrol: 24.1±2.4%, n = 91; 25 µM resveratrol: 21.3±0.7%, n = 89).

### Resveratrol prevents MG from activating the SAC protein BubR1

**Figure 7:**
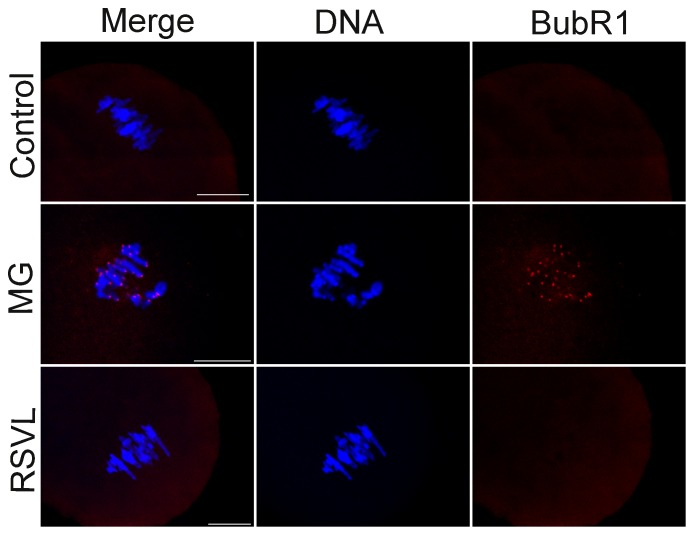
Effect of MG and resveratrol treatment on the activity of SAC protein BubR1. Detection of BubR1 in oocytes in control group, MG-treated group and RSVL-MG-treated group. Red, BubR1; blue, DNA. Scale bar  = 20 µm.

Stable kinetochore-microtubule (K-MT) attachment is critical for the correct chromosome segregation and unattached or improperly attached chromosomes would activate the spindle assembly checkpoint (SAC) pathway. Since a lack of stable chromosome arrangement was observed in the above experiments, we assessed the localization of BubR1 in oocytes from MG and resveratrol group, which is a key component of SAC proteins. Specific signals for BubR1 were detected in the MI arrest oocytes in the MG group, while the control and resveratrol with MG group showed no signals of BubR1 (**Fig. 7**). This result suggested that the MG-inducing MI arrest may be related to the activation of BubR1 and resveratrol could reverse this progress.

## Discussion

Toxic MG and its derivative are supposed to accumulate and play a role in reducing quality and developmental potential of mammalian oocytes of aged and diabetic females[10], [32]. Previous studies have shown that MG induces DNA damage, spindle aberrations, lack of mitochondrial integrity and subsequent apoptosis in oocytes[10], [11]. In this study, we performed several biological assays related to the oxidative damage, and found that the antioxidant resveratrol could protect oocytes from the damage effect of MG. Our results indicated that resveratrol may present potential protective effects for oocytes, which also provided groundwork for a strategy combatting the long-term exposure of oocytes to supra-physiological MG concentrations in diabetes and ageing females.

### Resveratrol scavenges ROS induced by MG in oocytes

It has been reported that, as a representative metabolite in diabetes and ageing, MG contributes to excessive ROS in oocytes and subsequently affects the oocyte quality. ROS are produced within the follicle during the ovulatory process and oxidative stimulation has important physiological roles which promote oocyte maturation and follicular wall rupture within the follicle[33]. However, under pathological conditions, the excessive ROS may contribute to oxidative stress. Scientific endeavors have been focusing attention on ROS as an important role associating diabetes, ageing and other diseases with poor oocytes. MG can generate ROS induced by autoxidation[27]. Besides, MG could further modify some enzymes such as superoxide dismutases, glutathione peroxidases and glutathione transferases which result in a decreasing antioxidant defense way[28]. Through interaction with large molecules, MG produces various toxic substances which are so called AGEs and then cumulatively lead to more cellular oxidative stress[5].

Recently, people focused on the protective antioxidant effect of resveratrol to the female reproductive system. Previous studies demonstrated that resveratrol treatment during IVM had beneficial effects on porcine oocyte maturation and subsequent embryonic development[34], [35]. Moreover, resveratrol protected against the reduction of fertility ability of mice with aging[36]. All of these protective effects are related to the abilities of resveratrol to eliminate the cellular excessive ROS in both oocytes and embryos. In our study, resveratrol scavenged ROS induced by MG (**Fig. 4** **and Table. 1**). We considered it as the essence of the protective effect of resveratrol to the MG-induced damage to oocytes.

### Resveratrol could reduce DSBs contributed by MG

DSBs can be induced by endogenous metabolites and exogenous stimulations such as ROS[26]. Unlike programmed DNA damage, the ROS-induced abnormal DSBs of cellular DNA, if not repaired immediately, can induce chromatin remodeling, cell cycle arrest, cell cycle delay, apoptosis or other forms on cell death[37], [38]. DNA strand breakage could be induced by the ROS generated from the reaction of MG with amino acids[39]. It has been reported that antioxidant genistein protected MG-induced oxidative DNA damage and cell injury in human mononuclear cells[40]. The results were similar to ours, suggesting that a supplement of antioxidants may prevent the formations of DSBs. In fact, there are a few of spontaneous DSBs occurring at the pachytene stage in oocytes. DSB lesions in this phase can be repaired by homologous recombination during which homologous chromosomes form synaptonemal complexes. However, an excess of DSBs induced by ROS can not be reduced by activating the DNA repair systems and this often results in a lower maturation rates. In present study, we considered that DSBs occurred in oocytes those were with more than tenγ-H2AX positive foci per nucleus and resveratrol could reduce the frequency of such occurrences of DSBs in oocytes (**Fig. 1**). Similarly, TUNEL-positive staining of blastocysts demonstrated that MG could disturb the DNA integrity, and this effect could be blocked by resveratrol[41]. Both results supported the notion that resveratrol could protect DNA integrity affected by MG which may contribute to other cytotoxic effects.

### Effects of Resveratrol and MG on the transition of MI to MII stage

It is known that assembly of meiotic spindle and chromosome congression in MI stage are crucial for female gamete formation and failure of those processes could result in cell cycle arrest[42]. Resveratrol could block MG disturbing the accurate chromosome alignment which is important for correct K-MT attachments and chromosome separation, both of which ensure polar body extrusion and euploid gamete formation. ROS could induce a failure of chromosome congression with evidence stemming from exposures to tert-butyl hydroperoxide (tBH) during IVM. Tert-butyl hydroperoxide resulted in changes in spindle morphology concomitant with defects in chromosome alignment and a higher incidence of aneuploidy[43]. Likewise, antioxidant supplements in food could result lowered incidence of aneuploidy in oocytes[44], which supported our notion that a supplement of antioxidant would counteract exogenous oxidative damage on oocytes.

The SAC is essential for the oocyte's transition from MI to anaphase I (AI). Those proteins including Mad2, Bub1, Bub3 and BubR1 ensure correct segregation of homologous chromosomes and provoke a cell cycle arrest in metaphase if any chromosome is not correctly attached to the bipolar spindle[45]. When the SAC function is blocked, K-MT attachments are no longer surveillanced[45]. Chromosomes misaligned in BubR1-depleted oocytes suggested the importance of this surveillance system which make sure that microtubules can encounter kinetochores and not soon be depolymerized[46]. In this case, we found that treatment with MG resulted in decrease of PBE rate, failure of spindle assembly and misaligned chromosomes (**Fig. 2** **,** **3**). So we detected BubR1 in MG-treated oocytes and found that BubR1 was activated. While in resveratrol-treated group we did not detect any signal of BubR1 (**Fig. 7**). This result confirmed that MG may affect K-MT attachment and activated the SAC protein BubR1, and this affect could also be alleviated by resveratrol in mouse oocyte meiosis.

### Resveratrol, MG and abnormal mitochondrial function

During oocyte maturation, the mitochondrial distribution plays a primary role in energetic metabolism, homeostasis, and cell death[47]. Decreased ATP supply and ROS may lead to abnormal oocyte mitochondrial redistribution and the formation of large heterogeneous mitochondrial fragmentation, and thus insufficient energy supplied to the nuclei and other organelles, which may be a cause for the delay or an arrest of oocyte development.

Immunofluorescent microscopy analysis revealed that MG leads to the inadequate translocation of mitochondria during oocyte maturation. One conspicuous tendency was that the percentage of the perinuclear distribution pattern at GV stage and polarized distribution pattern at MII stage was decreased relative to control whereas the proportion of the homogenous distribution pattern was increased accordingly. In addition, at both GV and MII stages, oocytes treated with MG displayed a much higher percentage of clustering mitochondrial distribution. In contrast, protective affects of resveratrol can ensure the correct spatial remodeling of mitochondria (**Fig. 5**). Given that the redistribution of mitochondria may allow maturing oocytes to cater to differing energy requirements of various key events, such as germinal vesicle breakdown and metaphase spindle formation and rotation, a prevention effect of resveratrol to the inadequate translocation of mitochondria perhaps offer assistance to overcome maturation delay and spindle defects.

### Resveratrol alleviates MG-induced lipid peroxidation

In most of the studies on oocyte and early embryo, the role of lipid has been overlooked. In fact, lipids such as fatty acids have numerous biological functions involved in energy metabolism and cell signal transduction[48]. Maintenance of the fusibility of oolemma to ensure accomplishment of meiosis and fertilization of oocyte is thought to be a remarkable function of lipid. However, once attacked by ROS, supraphysiological lipid peroxidation occurred which is thought to be a threat to change plasma membrane fluidity. Smoking constituent benzopyrene impaired oocyte fusibility through oxidative stress and this was mainly due to the lipid peroxidation[49]. Xenobiotic had the same effect[50]. In fact, the excessive ROS can almost result in lipid peroxidation. In our study, we used the lipid peroxidation probe BODIPY and found that resveratrol could decrease the level of lipid peroxidation induced by MG (**Fig. 6**). Given the integral role of lipid in formation and function of membrance, it was also probable that the aberrations induced by MG and the protective effect of resveratrol reported previously reflected changes in the profiles or integrity of lipid.

**Figure 8:**
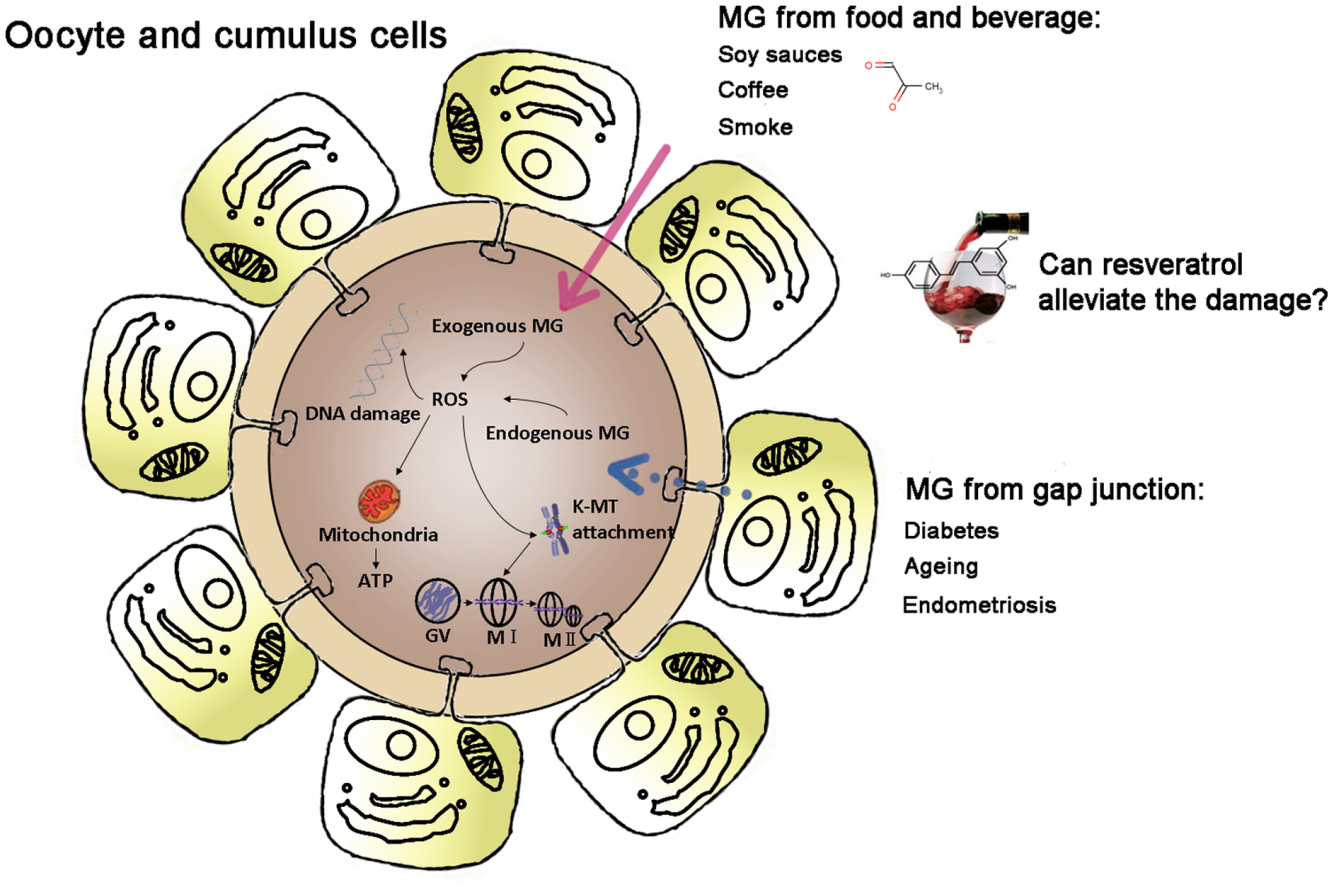
A schematic diagram showing that resveratrol could alleviate MG-induced cytotoxicity in oocytes.

In the present study, we showed that resveratrol could play an important role in promoting oocyte quality by preventing MG-induced DNA damage, decrease of PBE rate, abnormal mitochondrial distribution and lipid peroxidation in oocytes (**Fig. 8**). High level of serum MG and intracellular concentration is closely related to ageing and diabetes. Exogenous MG also exists in diet and water which may become a hazard to oocytes. Our findings may have clinical implications for the treatment of decreased quality oocytes exposed in endogenesis and exogenous MG. Future work should focus on the deep mechanism of the protective effects of resveratrol to the MG-inducing damage such as the alteration of proteome and cellular signal transduction.
